# Prediction of HIV drug resistance from genotype with encoded three-dimensional protein structure

**DOI:** 10.1186/1471-2164-15-S5-S1

**Published:** 2014-07-14

**Authors:** Xiaxia Yu, Irene T Weber, Robert W Harrison

**Affiliations:** 1 Department of Computer Science, Georgia State University, 34 Peachtree Street, Atlanta, GA 30303, USA; 2 Department of Biology, Georgia State University, Petit Science Center, Atlanta, GA 30303, USA

## Abstract

**Background:**

Drug resistance has become a severe challenge for treatment of HIV infections. Mutations accumulate in the HIV genome and make certain drugs ineffective. Prediction of resistance from genotype data is a valuable guide in choice of drugs for effective therapy.

**Results:**

In order to improve the computational prediction of resistance from genotype data we have developed a unified encoding of the protein sequence and three-dimensional protein structure of the drug target for classification and regression analysis. The method was tested on genotype-resistance data for mutants of HIV protease and reverse transcriptase. Our graph based sequence-structure approach gives high accuracy with a new sparse dictionary classification method, as well as support vector machine and artificial neural networks classifiers. Cross-validated regression analysis with the sparse dictionary gave excellent correlation between predicted and observed resistance.

**Conclusion:**

The approach of encoding the protein structure and sequence as a 210-dimensional vector, based on Delaunay triangulation, has promise as an accurate method for predicting resistance from sequence for drugs inhibiting HIV protease and reverse transcriptase.

## Background

**Figure 1:**
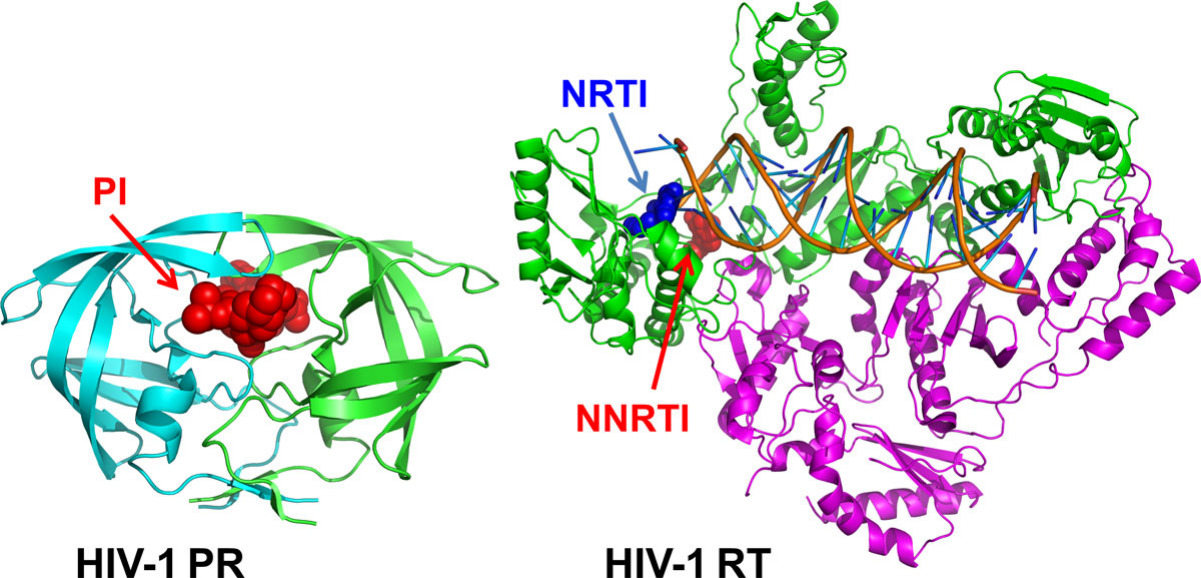
**Crystal structures of HIV-1 PR and RT**. The structure of HIV-1 PR dimer in complex with the inhibitor (PI) saquinavir is shown from50. The two subunits of HIV-1 PR are shown in green and cyan. The PI is colored red. The structure of HIV-1 RT dimer is shown in complex with DNA and bound NNRTI nevirapine (NVP) and NRTI zidovudine (AZT) from 51-52. The p66 subunit is shown in green and the p51 subunit is shown in purple. NRTI is shown in blue, and NNRTI is red. Double stranded DNA is indicated in orange.

HIV/AIDS is a pandemic disease and more than 30 million people are infected worldwide [1]. There is no effective vaccine or medicine to completely cure AIDS; however, the long-term survival of many patients has been enabled by drug therapy. Highly Active Antiretroviral Therapy (HAART) using three or four different drugs with different viral targets is very effective in stabilizing the infection [2]. These antiviral drugs target different stages in the viral life-cycle. Two important drug targets are the HIV protease (PR) and reverse transcriptase (RT), which have essential roles in viral replication. HIV RT converts the viral RNA genome into DNA, which is translated by the host cell machinery into the viral precursor proteins. HIV PR functions to cleave the large viral precursor proteins into individual enzymes and structural proteins, which produces infectious viral particles. Among the 23 approved drugs in current clinical use, there are seven nucleoside RT inhibitors (NRTIs), four non-nucleoside RT inhibitors (NNRTIs), and eight PR inhibitors (PIs) [3]. The approved PIs were designed to bind in the active site of HIV PR, and prevent the processing of viral precursor proteins (Figure 1). NRTIs are chemical analogs of the natural nucleoside substrates of the HIV RT that bind to the protein active site and block its activity in synthesizing DNA from viral RNA. The inhibitors in the NNRTI class also decrease the enzymatic activities of RT, however, they bind in an allosteric site in the palm domain of the p66 subunit instead of the active site of RT (Figure 1).

Despite the success of HAART, current therapy is limited by the rapid emergence of drug resistance [3]. The virus can mutate to acquire resistance during therapy due to the lack of proofreading by RT [4] and high replication rate [5]. These resistance mutations alter the drug targets such as PR and RT [6]. Some of the 35 mutations associated with resistance to PRIs alter amino acids located in the active site of PR while the majority alter residues in distal regions of the enzyme structure [7]. Similarly for RT, several of the mutations associated with resistance to NRTIs alter amino acids in the active site of the enzyme while others are located in more distal regions. The amino acid mutations occurring in association with resistance to the NNRTIs tend to cluster around the inhibitor binding site [8,9]. The molecular mechanisms for these antiviral drugs are described in the review [10].

These resistance mutations lower the effectiveness of specific drugs and may cause failure of the treatment. Infections with resistant HIV are prevalent; surveys in North America and Europe show that 8-20% of HIV infections in untreated people contain primary drug resistance mutations [10]. Over time, multiple mutations can accumulate giving a huge number of possible combinations of mutations in each protein. This persistent problem led to the recommendation for resistance testing to guide the choice of drugs in AIDS therapy [11-13]. Fast sequencing of the genome of the infecting virus can be combined with computational predictions of resistance to guide the choice of effective antiviral drugs [13]. Accurate and fast computational predictions are desirable to avoid the expense, limited availability and time involved for performing an experimental cell-based assay for resistance where results can take four weeks.

Accurate predictions can be valuable for prescribing the most effective drugs for infections with resistant HIV. Most genotype interpretation algorithms in clinical use are knowledge based [14]. These interpretation algorithms apply a set of rules or scores for each mutation and drug. The performance of several commonly used interpretation algorithms: Stanford HIVdb [15], HIV-grade [16], REGA and ANRS (http://www.hivfrenchresistance.org/) has been compared [16]. In addition, many computational classification techniques have been evaluated for predicting drug resistance from the genotype data. The standard classification techniques of artificial neural networks (ANN) [17-21], decision tree [19-22], random forests [21], support vector machine (SVM) [21-23] and regression analysis [19] have been applied in HIV drug resistance predictions. Statistical methods can also be applied to analyze the relationship between genotype and phenotype. The association of mutations with resistance to the PIs saquinavir (SQV) and indinavir (IDV) was determined using cluster analysis, recursive partitioning, and linear discriminant analysis [24]. These methods are limited by the high dimensionality of the genotype data, hence non-parametric methods have been proposed and tested on resistance data for the PI amprenavir [25,26]. Protein structural information has been used to generate statistical potentials of mutants for training with SVM or random forest learning algorithms and tested in predicting resistance to the RT inhibitor nevirapine (NVP) [27].

We have evaluated an efficient encoding of information from the three-dimensional protein structure for the prediction of resistance from genotype. The structural encoding via Delaunay triangulation improves the quality of the predictions by representing interactions between amino acid neighbours in the three-dimensional structure unlike the linear sequence representation of other methods. This unified sequence-structure representation was used in supervised training with SVM, ANN, and a new sparse dictionary classification method. The compressive sensing/sparse dictionary representation [28,29] has been applied successfully in image analysis to enhance learning capacity and efficiency. Sparse representation has been employed for image restoration [30,31], denoising [32], deblurring [33], signal processing [34], and face detection [35]. Initial tests of this procedure for classifying resistance to four PIs was presented in [36]. Here, the structural encoding has been expanded to regression analysis and classification of genotype-phenotype data for seven PIs, six NRTIs and three NNRTIs.

## Results

We combined structural information with genotype for regression analysis and supervised learning on resistance data. The new graph based sequence-structure encoding was tested with the Genotype-Phenotype Data from the Stanford HIV drug resistance database [37] (http://hivdb.stanford.edu/cgi-bin/GenoPhenoDS.cgi). Data were available for two different protein targets: HIV-1 PR and HIV-1 RT. For HIV-1 PR, eight PR inhibitors atazanavir (ATV), IDV, nelfinavir (NFV), ritonavir (RTV), lopinavir (LPV), tipranavir (TPV) and SQV were tested. While for the study of HIV RT inhibitor resistance, NNRTIs nevirapine (NPV), delaviridine (DLV), efavirenz (EFV), and NRTIs lamivudine (3TC), abacavir (ABC), zidovudine (AZT), stavudine (D4T), didanosine (DDI) and tenofovir (TDF) were tested. The data include the protein sequence (genotype) and resistance value (phenotype) from the PhenoSense (ViroLogic™) assay for each virus isolate. Genotype-phenotype data were available for 744 to 1674 isolates for different inhibitors of HIV PR, while RT was represented by 353 to 746 records for the 9 different NRTIs and NNRTIs. The preprocessing of the sequence and resistance data is detailed in Methods. Genotypes were expanded to unique protein sequences due to the presence of more than one amino acid at some positions. This expansion resulted in a total of 10,228 to 17,545 unique sequences of HIV PR mutants and 2,004 to 11,367 RT mutants for the various inhibitor resistance values.

### Graph based protein sequence/structure representation using Delaunay triangulation

The sequences were combined with information from the three-dimensional protein structures by employing a graph generated by Delaunay triangulation as described in [38]. Two structure templates were used: 3OXC [4] for HIV-1 PR, and 2WOM [39] (from http://www.pdb.org). Only one structure vector is needed for each protein. In other words, all PR mutant sequences are combined with a single 210-dimensional vector derived from one PR structure, and similarly, a single structure vector is used for the RT mutants in subsequent regression and classification of resistance data. As a result, all mutants are represented as vectors of constant dimensionality, which is a desirable property for most of the pattern recognition algorithms. This structure vector was combined with sequences in regression analysis and classification for resistance.

### Multiple regression on HIV protease inhibitor resistance

**Figure 2:**
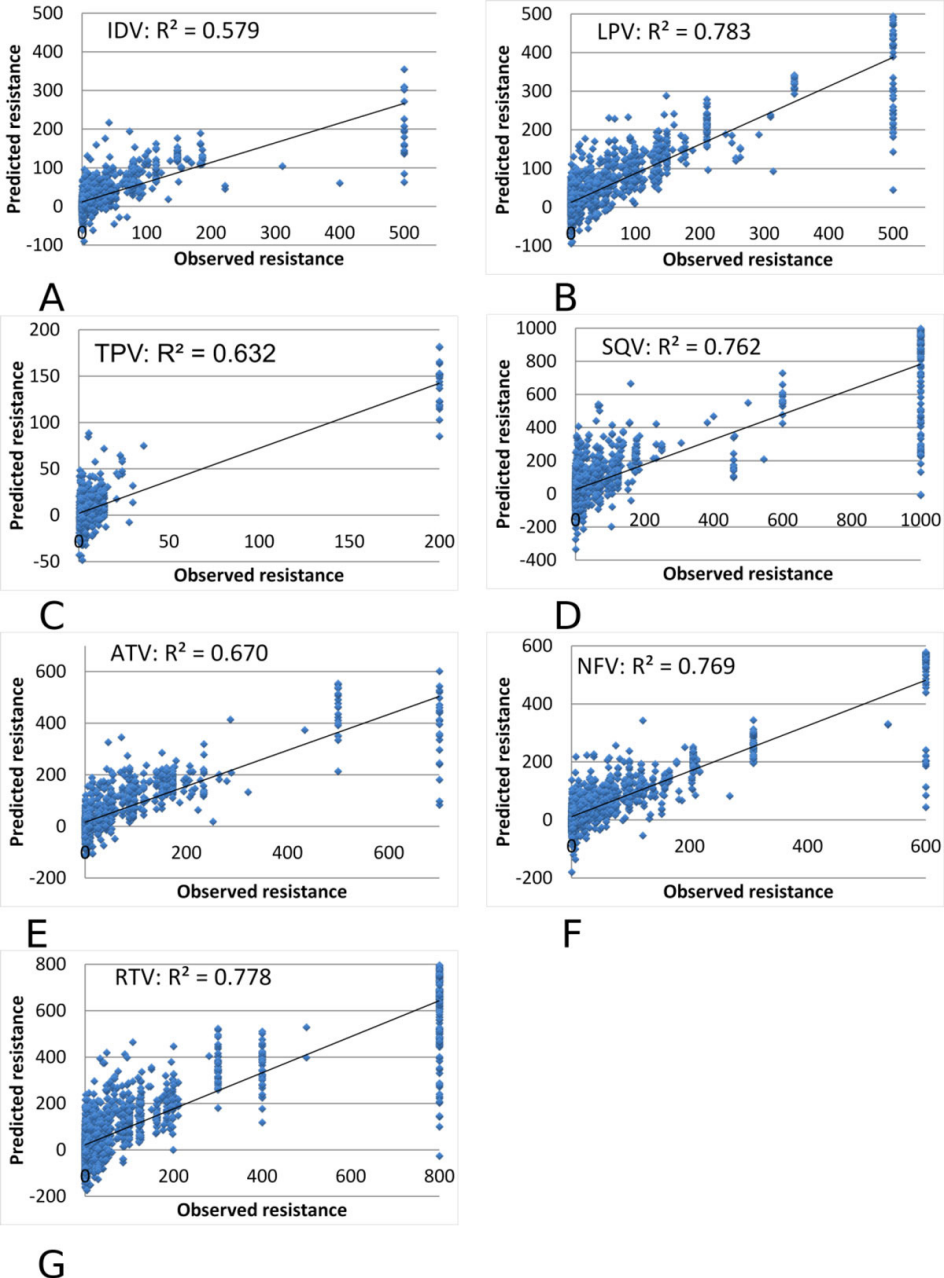
**Multiple regression on the predicted and observed resistance for HIV-1 PR inhibitors**. The predicted resistance is plotted against the observed value as blue dots. The observed resistance is measured relative to a value of zero for the standard non-resistant virus. The trend line is shown. The regression results are shown for resistance to PIs: **(A) **IDV, **(B) **LPV, **(C) **TPV, **(D) **SQV, **(E) **ATV, **(F) **NFV, and **(G) **RTV.

After each of the mutated sequences was represented by a 210-dimensional vector, we performed the regression analysis for the drug resistance data. We performed k-fold (k = 5) regression analysis on the sequence and resistance data. The predicted values for relative resistance are plotted against the experimental values as shown in (Figure 2) for the PR inhibitors ATV, NFV, RTV, IDV, LPV, TPV and SQV.

The multiple regression gave high R^2 ^values of 0.579-0.783 and very low standard deviations as listed in Table 1. The values are the average of all the R^2 ^values from k-fold regression. The high variance seen for high values of resistance is likely due to limitations of the experimental assay such that the measured resistance value has a cutoff at the upper limit, while the viral strains may have an effective resistance above this cutoff. The excellent correlations demonstrate that relative resistance to PIs can be predicted successfully from genotype by the new sequence/structure encoding method. In order to avoid training to an "optimal" n-fold set for cross validation, cross validation sets are chosen independently for each training run. Therefore, there is always a small variation in the results.

**Table 1:** Multiple regression on predicted relative resistance to HIV-1 PR inhibitors.

	IDV	LPV	TPV	SQV	ATV	NFV	RTV
R^2 ^values, mean	0.579	0.783	0.632	0.762	0.670	0.769	0.778

R^2 ^values, stddev	0.037	0.014	0.045	0.018	0.035	0.029	0.016

### Multiple regression on HIV reverse transcriptase inhibitor resistance

**Figure 3:**
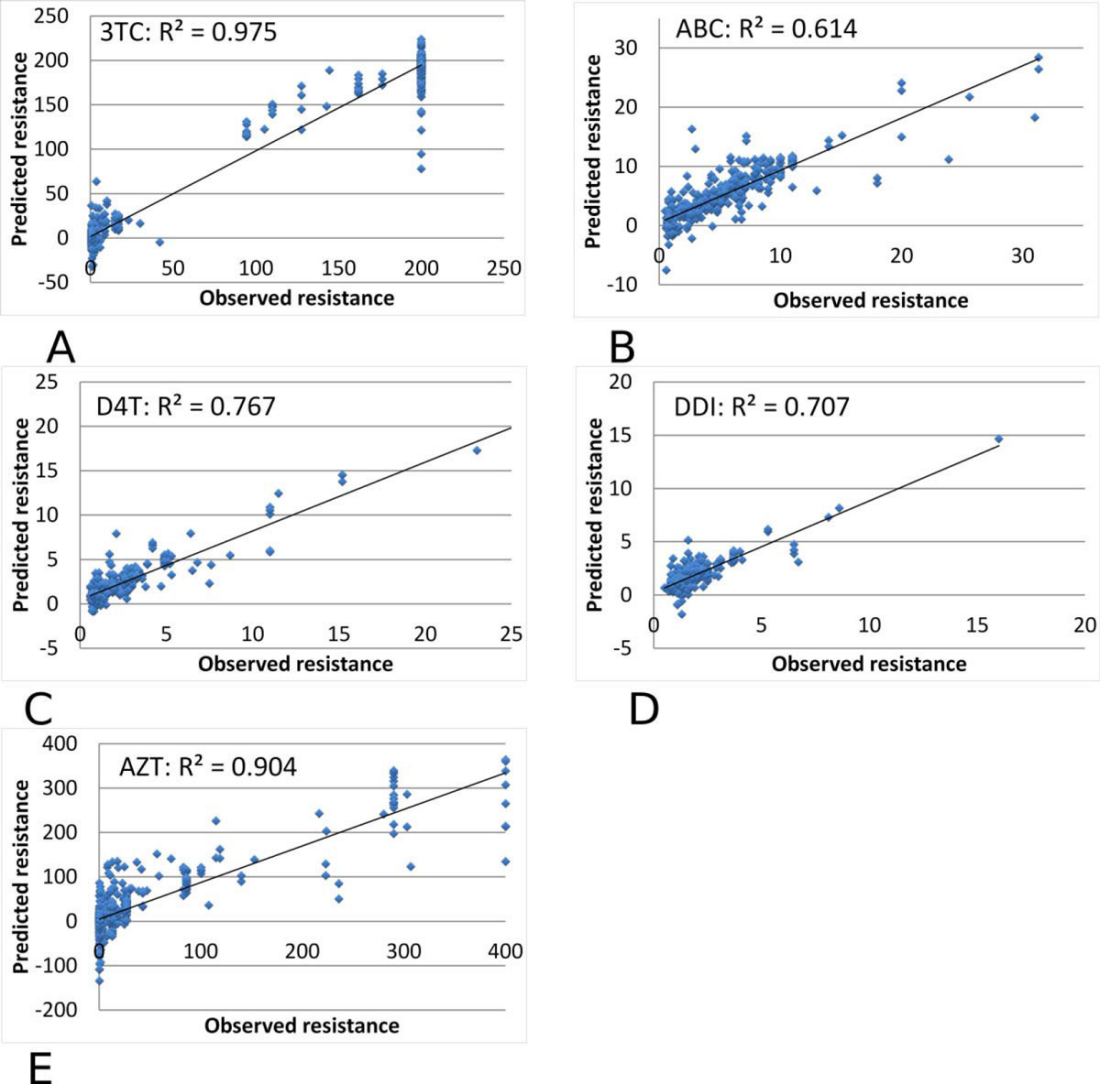
**Multiple regression on the predicted and observed resistance for HIV-1 NRTIs**. The predicted resistance is plotted against the observed value as blue dots. Observed resistance is measured relative to a value of zero for the standard non-resistant virus. The trend line is shown. The regression results are shown for resistance to NRTIs: (A) 3TC, (2) ABC, (3) D4T, (4) DDI, and (5) AZT.

**Figure 4:**
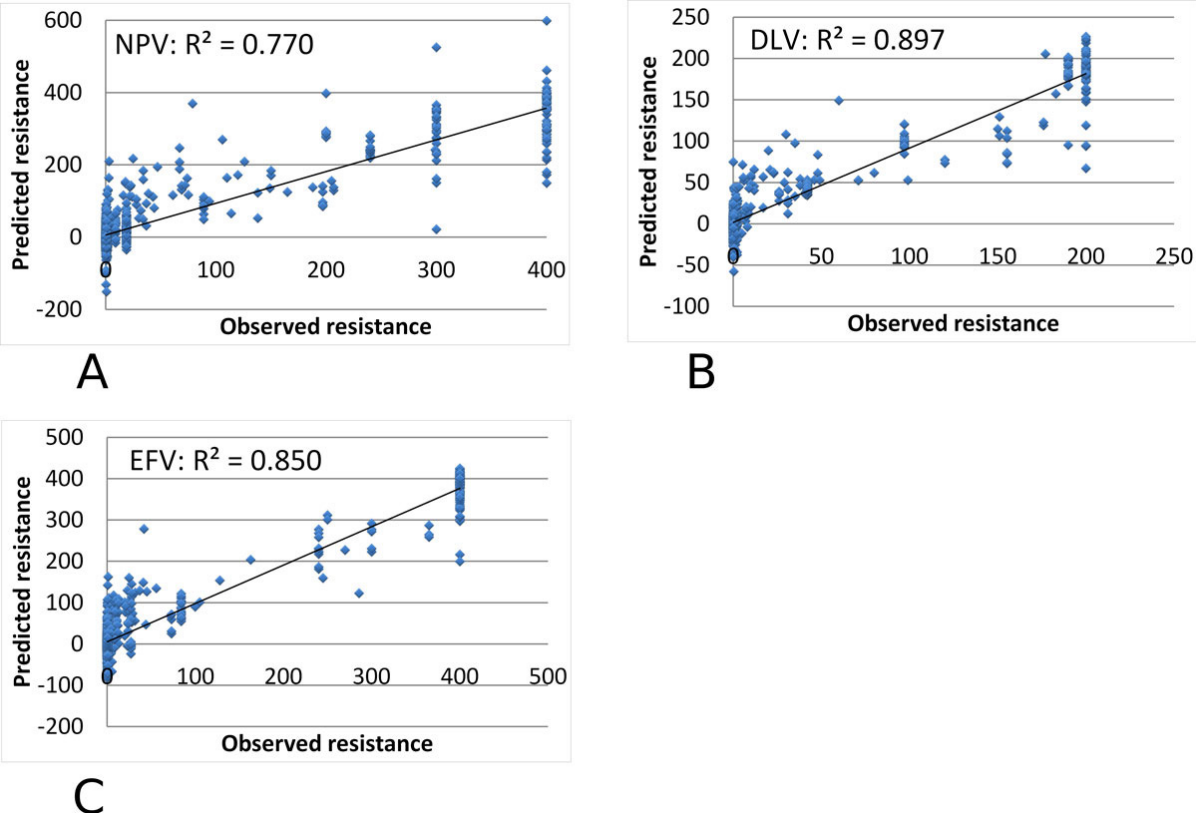
**Multiple regression on the predicted and observed resistance for HIV-1 NNRTIs**. The predicted resistance is plotted against the observed value as blue dots. Observed resistance is measured relative to a value of zero for the standard non-resistant virus. The trend line is shown. The regression results are shown for resistance to NNRTIs: (A) NPV, (B) DLV, and (3) EFV.

Multiple regression analysis was performed similarly on genotype-phenotype data for drugs inhibiting HIV-1 RT. The predicted and observed values are compared for resistance to NRTIs: 3TC, ABC, D4T, DDI, TDF and AZT in Figure 3; and NNRTIs: NPV, DLV and EFV in Figure 4.

The regression results gave high R^2 ^values of 0.614-0.975 for the different RT inhibitors, as shown in Tables 2 and 3. The resistance to NRTIs was predicted with excellent R^2 ^values of 0.85-0.90 and very low standard deviations, while resistance predictions for NRTIs gave R^2 ^values in the larger range of 0.61-0.98. Larger standard deviations were obtained for analysis of ABC and DDI possibly because the range of values in the dataset was smaller than for the others. Therefore, graph based encoding had excellent success in predicting resistance to RT inhibitors.

**Table 2:** Multiple regression on predicted relative resistance for NNRTIs.

	DLV	EFV	NPV
R^2 ^values, mean	0.904	0.897	0.850

R^2 ^values, stddev	0.015	0.012	0.015

### Classification of resistance with support vector machine

The support vector machine (SVM) was proposed by Vapnik [40], and is widely used as a supervised learning classifier in the machine learning classification area. In this experiment, 5-fold cross validation tests were performed by implementing in MATLAB SVM toolbox [41,42] and the linear kernel was used. The results are shown in Tables 3, 4, 5 for HIV-1 PR inhibitors (PIs), HIV-1 RT NRTIs and HIV-1 RT NNRTIs. This classification shows high accuracy, sensitivity and specificity for all inhibitors. For PIs the accuracy values range from a low of 0.93 to a high of 0.96, while sensitivity and specificity range from 0.92-0.96 and 0.94-0.98, respectively. Resistance to NRTIs is classified with even higher accuracies of 0.97-0.99, sensitivities of greater than 0.98 and specificities of 0.95-0.99, while for NNRTIs the classification performance was superior with all values of over 0.97 for accuracy, sensitivity and specificity. The excellent performance with the linear SVM kernel demonstrates conclusively that the novel encoding using Delaunay triangulation separates the resistant and non-resistant data into two distinct categories.

**Table 3:** Multiple regression on predicted relative resistance for NRTIs.

	AZT	3TC	ABC	D4T	DDI
R^2 ^values, mean	0.770	0.975	0.614	0.767	0.707

R^2 ^values, stddev	0.023	0.004	0.253	0.061	0.146

**Table 4:** Classification using SVM for Resistance to PIs.

	ATV	IDV	NFV	RTV	LPV	SQV	TPV
Accuracy	0.955	0.960	0.933	0.946	0.962	0.946	0.961

Stddev (×10^2^)	0.400	0.510	0.350	0.580	0.220	0.580	0.290

Sensitivity	0.943	0.951	0.923	0.945	0.952	0.945	0.957

Stddev (×10^2^)	0.600	1.00	0.400	0.910	0.270	0.910	0.410

Specificity	0.968	0.970	0.943	0.947	0.972	0.947	0.965

Stddev (×10^2^)	0.450	0.290	0.820	0.890	0.280	0.890	0.410

**Table 5:** Classification using SVM for Resistance to NRTIs.

	3TC	ABC	AZT	D4T	DDI	TDF
Accuracy	0.987	0.981	0.984	0.992	0.965	0.975

Stddev (×10^2^)	0.484	0.234	0.390	0.371	0.289	0.914

Sensitivity	0.984	0.981	0.984	0.991	0.977	0.979

Stddev (×10^2^)	0.613	0.379	0.627	0.417	0.436	1.21

Specificity	0.991	0.982	0.984	0.993	0.954	0.970

Stddev (×10^2^)	0.510	0.397	0.470	0.505	0.625	1.76

### Classification with Artificial Neural Networks

As in the SVM experiment, the 5-cross validation test was applied to the Artificial Neural Networks (ANN) to classify genotype-phenotype data for resistance. Specifically, the three-layer feedforward network was used in Matlab [42-44]. The network had one hidden layer of 20 nodes and was trained with backpropagation with a maximum of 50 training epochs. The results are shown in Tables 6, 7, 8 for HIV-1 PR inhibitors, and RT inhibitors NRTIs and NNRTIs. The values calculated for accuracy, sensitivity and specificity for resistance to PIs have a low of 0.91 and reach 0.97. Improved performance was achieved for classifying resistance to RT inhibitors compared with PIs. Results for NRTIs gave values of accuracy, sensitivity and specificity of 0.96-0.99, while for NNRTIs all values were greater than 0.98.

**Table 6:** Classification using SVM for Resistance to NNRTIs.

	NPV	DLV	EFV
Accuracy	0.982	0.983	0.991

Stddev (×10^2^)	0.254	0.473	0.316

Sensitivity	0.972	0.976	0.986

Stddev (×10^2^)	0.490	0.600	0.618

Specificity	0.992	0.991	0.996

Stddev (×10^2^)	0.397	0.787	0.301

**Table 7:** Classification using ANN for Resistance to PIs.

	ATV	IDV	NFV	RTV	LPV	SQV	TPV
Accuracy	0.958	0.944	0.917	0.934	0.963	0.957	0.951

Stddev (×10^2^)	0.320	1.25	1.38	1.44	0.641	0. 723	1.27

Sensitivity	0.959	0.940	0.913	0.935	0.965	0.958	0.953

Stddev (×10^2^)	0.460	1.56	2.46	1.13	0.741	0.483	1.89

Specificity	0.957	0.947	0.922	0.933	0.961	0.956	0.950

Stddev (×10^2^)	0.440	0.944	1.05	1.97	0.598	1.06	0. 672

**Table 8:** Classification using ANN for Resistance to NRTIs.

	3TC	ABC	AZT	D4T	DDI	TDF
Accuracy	0.982	0.984	0.987	0.983	0.965	0.970

Stddev (×10^2^)	0.469	0.525	0.164	0.452	0.176	1.21

Sensitivity	0.984	0.978	0.988	0.980	0.973	0.965

Stddev (×10^2^)	0.994	0.700	0.428	0.983	0.434	1.67

Specificity	0.980	0.991	0.986	0.986	0.957	0.975

Stddev (×10^2^)	0.835	0.474	0.490	0.687	0.168	1.00

### Classification using sparse dictionary

The sparse dictionary classifier was also implemented using the 5-fold cross validation tests using the approach described in [36]. The results are shown in Tables 7, 8, 9 for HIV-1 PR inhibitors, HIV-1 RT NRTIs and NNRTIs. High values were obtained for accuracy, sensitivity, and specificity. Accuracies ranged from 0.95-0.99 for resistance to PIs, 0.82-0.92 for NRTIs and 0.81-0.84 for NNRTIs. The sensitivities were all greater than 0.93 for the calculations on resistance to PIs, and specificities were greater than 0.96. Lower values were obtained for calculations on some of the RT inhibitors where values for sensitivity ranged from 0.75 to 0.96, while high specificity values from 0.86 to 1.00 was calculated. These performance measures are somewhat poorer than for the standard SVM and ANN classifiers. It is not surprising; however, that more development may be necessary for applying the new sparse dictionary as a classifier since previously it has been employed primarily for image processing.

**Table 9:** Classification using ANN for Resistance to NNRTIs.

	NPV	DLV	EFV
Accuracy	0.983	0.986	0.986

Stddev (×10^2^)	0.524	0.488	0.503

Sensitivity	0.979	0.985	0.982

Stddev (×10^2^)	0.507	1.24	0.955

Specificity	0.987	0.987	0.990

Stddev (×10^2^)	0.554	0.448	0.462

### Comparison with standard genotype interpretation methods

Finally, we compared our methods with the standard drug resistance prediction methods HIV-GRADE, ANRS-rules, Stanford HIVdb, and Rega, which are available at http://www.hiv-grade.de/cms/grade/, using the same genotype-phenotype datasets described in Methods. The procedure discussed in [36] was used to convert the protein sequences into nucleotide sequences. Other methods usually give resistance interpretations in three categories of "resistance, "intermediate" and "susceptible". Since multiple classification is difficult with SVM and ANN, only two classes were considered for calculating the accuracy. Both "resistant" and "intermediate" are considered as "resistant"; while "susceptible" is considered as "non-resistant". The results are shown in Tables 10, 11, 12 for HIV-1 PR inhibitors, HIV-1 RT NRTIs and NNRTIs. N/A means that no output was obtained from the server for this dataset.

**Table 10:** Classification using sparse dictionary for resistance to PIs.

	ATV	NFV	RTV	IDV	LPV	SQV	TPV
Accuracy	0.973	0.946	0.962	0.969	0.974	0.970	0.990

Stddev (×10^2^)	0.262	0.602	0.269	0.151	0. 292	0.139	0.277

Sensitivity	0.961	0.927	0.968	0.951	0.957	0.959	0.984

Stddev (×10^2^)	0.244	0.635	0.976	0.529	0.494	0.604	0.423

Specificity	0.986	0.967	0.958	0.989	0.992	0.981	0.995

Stddev (×10^2^)	0.661	1.44	1.23	0.297	0.361	0.692	0.199

**Table 11:** Classification using sparse dictionary for resistance to NRTIs.

	3TC	ABC	AZT	D4T	DDI	TDF
Accuracy	0.918	0.915	0.932	0.879	0.816	0.852

Stddev (×10^2^)	3.44	3.14	4.20	5.06	7.63	7.20

Sensitivity	0.963	0.872	0.947	0.814	0.801	0.789

Stddev (×10^2^)	2.60	5.08	4.73	6.81	6.11	8.45

Specificity	0.888	0.973	0.933	0.987	0.860	0.972

Stddev (×10^2^)	6.78	0.185	8.75	1.02	12.1	4.19

**Table 12:** Classification using sparse dictionary for resistance to NNRTIs.

	NPV	DLV	EFV
Accuracy	0.826	0.844	0.811

Stddev (×10^2^)	2.46	2.49	6.43

Sensitivity	0.761	0.773	0.753

Stddev (×10^2^)	3.48	3.82	8.43

Specificity	0.938	0.973	0.935

Stddev (×10^2^)	2.87	2.11	3.55

The accuracies demonstrate that classification with our structural encoding significantly outperforms other state of the art methods for predicting resistance to PIs for the three tested classifiers SVM, ANN and the sparse dictionary. Accuracies of 93.4-99.0% were obtained with structural encoding compared to 59.7-87.0% for the standard methods. The highest accuracies of greater than 95% were achieved with the sparse dictionary method. The prediction accuracy for resistance to the NRTI class of RT inhibitors also showed the advantages of our structural encoding with values of 81.6-99.2% compared with 72.7-95.9% for standard methods. In this case, the SVM and ANN classifiers performed better than the new sparse dictionary giving accuracies of at least 97%. For the NNRTIs, the structural encoding with SVM or ANN gave higher accuracies of 98.3-99.1% compared with 94.8-98.7% for standard methods. The sparse dictionary, however, showed lower performance with accuracies of 81.1-84.4% for NNRTI resistance, indicating some improvements may be needed for the new classifier.

## Discussion

The serious problem of drug resistance arising during therapy of HIV-infected individuals can be tackled by sequencing the HIV drug targets to identify mutations followed by computational prediction of resistance to guide the choice of effective therapy. Computational predictions of the most effective drugs for the mutated HIV provide a major advantage of low cost and speed relative to experimental assays for resistance. Most standard prediction methods are knowledge based methods, such as the genotype interpretation algorithms. These algorithms either use a set of rules, for example, the Visible Genetics/Bayer Diagnostics genotype interpretation rules [45], to generate the susceptibility of the infecting virus for each drug; or apply a score or 'penalty' for each drug such as the Stanford HIV database [46] and mutation rate based score [47]. Also, a combined rule-based and penalty-based method has been proposed and applied to both HIV-1 PR and RT inhibitors [48]. Although these methods are fast, they suffer from the major disadvantage of relying on specific known mutations strongly associated with resistance and cannot identify newly appearing resistance mutations, or assess the effects of many mutations more weakly associated with resistance.

Various machine learning and statistical methods have been applied to this problem, including the widely used classifiers, ANN [17,18], decision tree [22], and SVM [23]. Statistical methods such as cluster analysis, recursive partitioning, and linear discriminant analysis have been evaluated [24], and non-parametric methods proposed for high dimensionality data [25,26]. Most of these methods are based on the linear protein sequence and omit potentially valuable information from the three-dimensional protein structure. Additional information has been introduced in the form of 544 physicochemical descriptors for the amino acid mutations leading to correlation coefficients of 0.75-0.94 [20]. Other groups have included structural features such as PR-drug contacts in the binding site with majority voting [18]. In another example, Delaunay triangulation of the RT structure was combined with a four-body statistical potential derived from 1200 protein structures in predictions for resistance to NVP and gave cross-validated accuracies of 85% with SVM and 92% with random forest classifiers [27]. Molecular mechanics calculations on the PR-drug structure have been used to predict resistance of mutants, and gave high correlation (R2 of 0.76-0.85) between caclulation and IC50 from the experimental assay [51]. However, these calculations must be performed for each mutant-drug combination and will be slow for assessing large numbers of mutants for resistance.

We have developed a simple graph representation of protein structure for fast classification. The protein structure is a three-dimensional object that has many physical and chemical factors potentially effecting stability and activity. Previously, we showed that Delaunay triangulation was the best of several tested graph-based encodings of protein structure and sequence [37]. The graph-based encoding algorithm condenses a complicated three-dimensional object, a protein structure, into a relatively small hash function with 210 unique values per sequence and structure. One critical outcome is that the graph-based encoding results in a linearly separable data set that can be used readily by several different machine learning algorithms. Similarly, the encoding is sufficiently linear that straightforward multiple linear regression can be performed on the training data. The hash value maintains enough information about the complicated object to provide useful information for machine learning and regression.

This unified sequence-structure encoding gave high accuracy in initial tests on four PIs [36]. Here, we demonstrate successful application of the structure vector in multiple regression analysis and classification on resistance data for seven inhibitors of HIV PR and nine inhibitors of RT. The 5-fold validated regression analysis gave excellent correlation between predicted and observed resistance with excellent R2 values of 0.58-0.78 for PIs, 0.61-0.98 for NRTIs and 0.85-0.90 for NNRTIs. Classification with SVM, ANN or a new sparse dictionary method gave high accuracies for predicting the resistance for PR and RT inhibitors. The structure vector encoding had superior accuracy to predictions on the same sequences using standard interpretation algorithms. The sparse dictionary classifier was the best of tested classifiers for prediction of resistance to PIs, whereas SVM classification gave the best performance on resistance prediction for RT inhibitors. This structure vector encoding of genotype data has the advantage of using a single 210-dimensional vector for each protein target. The algorithm has one slow step for preparing the encoding from a single protein structure that can be applied to all genotypes in a fast calculation, in contrast to molecular mechanics calculations that must be set up in a non-trivial manner for each individual protein sequence. The entire protein sequence is combined with the structure vector, so there is the potential for accommodating new mutations or combinations of mutations with weak but concerted effects on resistance. The procedure can be extended easily in future calculations for resistant mutants with insertions in the protein sequence, which occur commonly in RT [3]. The new sparse dictionary classification approach can be extended to multiple classifiers by using more than two dictionaries, which is a significant advantage over the tested standard SVM or ANN classifiers, and may permit accurate predictions for different levels of resistance.

## Conclusions

The simple unified encoding of structural information with genotype gives high accuracy for prediction of resistance to HIV PR and RT inhibitors as well as excellent correlation coefficients in regression analysis. The improvement over algorithms using only linear sequence information suggests the importance of local interactions between mutated residues in the protein structure, which is consistent with the correlated local changes observed in the crystal structures of a highly resistant PR mutant with 20 substitutions [49]. Graph-based encoding of sequence and structure holds promise for fast and accurate predictions of resistance from sequence in order to guide the choice of effective drugs for treatment of HIV infections. In future, this approach can be expanded to predict resistance for other drugs and more diverse types of data.

## Materials and methods

### Data sets and data preparation

All the datasets were retrieved from Genotype-Phenotype Data on the Stanford HIV drug resistance database [37] (http://hivdb.stanford.edu/cgi-bin/GenoPhenoDS.cgi). In this experiment, the proposed algorithm was tested on two different systems: HIV-1 PR and HIV-1 RT resistance data. For HIV-1 PR, eight PR inhibitors atazanavir (ATV), indinavir (IDV), nelfinavir (NFV), ritonavir (RTV), IDV, LPV, TPV and SQV were tested. While for the study of HIV RT inhibitor resistance, NNRTIs nevirapine (NPV), delaviridine (DLV), efavirenz (EFV), and NRTIs lamivudine (3TC), abacavir (ABC), zidovudine (AZT), stavudine (D4T), didanosine (DDI) and tenofovir (TDF) were tested.

For the drug resistance study on the HIV PR and HIV RT inhibitors, all the genotypes were expanded to individual unique amino acid sequences using the method discussed in [36]. This expansion was needed since the genotyping experiment resulted in more than one possible amino acid at several positions in each genotype, due to potential experimental error or existence of multiple viral sequences infecting one patient. For each of the HIV-1 PR inhibitors the results were: for the inhibitor IDV, a total of 16846 sequences were obtained from 1622 isolates; for the inhibitor LPV, a total of 16269 sequences were obtained from 1322 isolates; for the inhibitor TPV, a total of 10228 sequences were obtained from 744 isolates; for the inhibitor SQV, a total of 17118 sequences were obtained from 1640 isolates; for the inhibitor ATV, a total of 12084 sequences were obtained from 1012 isolates; for the inhibitor IDV, a total of 16846 sequences were obtained from 1621 isolates; for the inhibitor NFV, a total of 17545 sequences were obtained from 1674 isolates; and for the inhibitor RTV, a total of 16652 sequences were obtained from 1589 isolates.

For each of the HIV-1 RT inhibitors the results were: for the inhibitor NPV, a total of 11367 sequences were obtained from 746 isolates; for the inhibitor DLV, a total of 11299 sequences were obtained from 732 isolates; and for the inhibitor EFV, a total of 11354 sequences were obtained from 734 isolates; for the inhibitor 3TC, a total of 4850 sequences were obtained from 633 isolates; for the inhibitor ABC, a total of 4846 sequences were obtained from 628 isolates; for the inhibitor AZT, a total of 4847 sequences were obtained from 630 isolates; for the inhibitor D4T, a total of 4845 sequences were obtained from 630 isolates; for the inhibitor DDI, a total of 4849 sequences were obtained from 632 isolates; for the inhibitor TDF, a total of 2004 sequences were obtained from 353 isolates.

All positive and negative instances of a given mutant were removed from either training or testing dataset before the cross-validation. This may avoid the potential problem of having negative instances associated with a positive test item or positive instances associated with a negative test item, and thus assure the training accuracy.

### Pre-processing of the datasets

In order to unify the data in the original datasets, those sequences with an insertion, deletion, or containing a stop codon relative to the consensus have been removed so that the data represent proteases of 99 amino acids.

Many of the sequence records in the dataset have multiple mutations at the same sites yet share the same drug-resistance value, which may be due to sequencing limitations or to the existence of multiple viral strains in the same isolate. In order to represent a single amino acids sequence for each mutant protein, we need to expand the data to multiple sequences with single amino acids at each location. For instance, in one 99-amino acid mutant of HIV PR, at one site there are two different types of amino-acids, and another site has three. In this case, this record must be expanded to a total of 6 = (2 × 3) different sequences, each of which has only one amino-acid for each of its 99 residues, sharing the same drug resistance. We designed a fast way to perform this expansion as detailed in [36], which significantly enriches the test data.)

### Cutoffs for resistance/susceptibility for each drug

For the HIV-1 PR inhibitors: ATV, IDV, NFV, and RTV, among all these genotype sequences, those mutants with the relative resistant fold < 3.0 were classified as non- resistant (susceptible), denoted as 0; while those with the relative resistant fold ≥ 3.0 were classified as resistant, denoted as 1 [19].

With the HIV-1 RT inhibitors: for ABC and TPV, those mutants with the relative resistant fold < 2.0 were classified as non-resistant, denoted as 0; while those with the relative resistant fold ≥ 2.0 were classified as resistant, denoted as 1; for 3TC, AZT, NPV, DLV, EFV, SQV, IDV and LPV those mutants with the relative resistant fold < 3.0 were classified as non-resistant, denoted as 0; while those with the relative resistant fold ≥ 3.0 were classified as resistant, denoted as 1; for D4T, DDI and TDF, those mutants with the relative resistant fold < 1.5 were classified as non-resistant, denoted as 0; while those with the relative resistant fold ≥ 1.5 were classified as resistant, denoted as 1 [19].

### Encoding structure and sequence with Delaunay triangulation

The sequence and structure of the protein were represented using a graph-based encoding as described in [36]. Delaunay triangulation was used to define a graph which spanned the protein structure and defined adjacent pairs of amino acid residues. Adjacent pairs of amino acids were summarized into a vector of the 210 unique kinds of amino acid pairs by calculating the distance for each adjacent pair in the structure and tabulating by the types of amino acids in that adjacent pair. Only the sequences of the mutated proteins are needed and only one protein structure is necessary. As a result, all mutants are represented as vectors of the same dimensionality, which is a desired property for most of the pattern recognition algorithms. The structures 3OXC [4] for HIV-1 PR, and 2WOM [39] for HIV-1 RT (from http://www.pdb.org) were used as templates for Delaunay triangulation.

### k-fold validation

In order to fully use all the data, a k-fold cross-validation was performed in all the experiments for all the drugs. Specifically, we randomly choose (*k*-1)/*k *of all the sequences (some are drug resistant, while others are non-drug resistant) for training the classifier and the remaining 1/*k *data are used for testing. These tests used *k *= 5. Independent randomly selected k-folds were chosen throughout the study to avoid bias in the results.

### Regression analysis for drug resistance prediction

The Genotype-Phenotype Datasets provide a drug resistance value, with respect to a certain type of drug, with each genotype. The mutations relative to a standard sequence are denoted as $x_{1},x_{2},\ldots ,x_{N};x_{i}\in {ℜ}^{210}$ where *N *is the total number of mutations and ${ℜ}^{210}$ is the structure vector. Also the corresponding drug resistance values are denoted as the real numbers $y_{1},y_{2},\ldots ,y_{N};y\in ℜ$ including 0 for the resistance value of the wild type virus. We then seek a linear model between the *x_i_*'s and *y_i_*'s by minimizing the cost function *E *:

(1) $$E:=\overset{N}{\underset{i=1}{\sum }}{\left(y_{i}-A\cdot x_{i}-b\right)}^{2}$$

with respect to the 210 dimensional vector A and scalar b.

Furthermore, in order to better utilize the available data set, we performed a *k*-fold cross-validation (in this work, k = 5). Specifically, the training set of size *N *is randomly divided into *k *groups. Among them, *k *- 1 groups are utilized for constructing the linear model as in Equation (1). Then, the linear model is used to predict the drug resistance for the remaining group with *N/k *mutations. The predicted resistances are compared with the measured ones and the R^2 ^values are recorded. Finally, the average and standard deviation of the *k *R^2 ^values are computed.

### Sparse dictionary classification

In this experiment, we applied our newly proposed method described in [36] on both HIV-1 PR and HIV-1 RT data sets. In this case, the sequences of the mutants are considered as the group of signals, and given these signals, we would like to construct a dictionary to represent them sparsely.

The construction of a dictionary can be considered as finding a suitable over-complete basis (frame), in which the signals of interest would be represented with far fewer non-zero coefficients, than in an arbitrary fixed basis such as a Fourier basis. The newly constructed basis is also called a dictionary. This dictionary can be used to assess how well the new signal fits the model represented by the dictionary, and therefore, it can be used as a new classification method.

In our experiment, we assume there are 2 groups of signals: one for drug resistant mutants, while the other group is non-drug resistant mutants. We construct two dictionaries for each group, respectively. After that, given a new signal (mutant, in our case), we use both dictionaries to represent this signal. By calculating and comparing the reconstruction error, the dictionary with the smaller error indicates that the signal belongs to this category. Based on the theory of the dictionary, it can be observed that the group number is not limited to 2, and such procedure could be used as a multi-group classification method. The 2 dictionaries for each set of drug resistance data were constructed and the classification performed as described in [36].

### List of Abbreviations

HAART, Highly Active Antiretroviral Therapy; PR, HIV protease; PI, protease inhibitor; RT, HIV reverse transcriptase; NRTI, nucleoside RT inhibitor; NNRTI, non-nucleoside RT inhibitor; ANN, artificial neural networks; SVM, Support Vector Machine; APV, amprenavir; ATV, atazanavir; IDV, indinavir; LPV, lopinavir; NFV, nelfinavir; RTV, ritonavir; SQV, saquinavir; TPV, tipranvir; 3TC, lamivudine; ABC, abacavir; AZT, zidovudine; D4T, stavudine; DDI, didanosine; DLV, delaviridine; EFV, efavirenz; NVP, nevirapine; TDF, tenofovir.

## Competing interests

The authors declare that they have no competing interests.

**Table 13:** Accuracy (%) compared to other methods for HIV-1 PR inhibitors.

	ATV	NFV	IDV	LPV	SQV	TPV
HIV-grade	84.7	81.2	85.1	80.5	80.2	72.8

ANRS	N/A	78.1	85.1	87.0	N/A	59.7

HIVdb	N/A	83.4	N/A	83.9	N/A	76.8

Rega	84.4	82.2	85.6	84.0	69.3	N/A

SVM	95.5	96.0	94.6	96.2	94.6	96.1

ANN	95.8	94.4	93.4	96.3	95.7	95.1

Sparse dictionary	97.3	94.6	96.9	97.4	97.0	99.0

**Table 14:** Accuracy (%) compared to other methods for HIV-1 RT NRTIs.

	3TC	ABC	AZT	D4T	DDI	TDF
HIV-grade	91.5	89.7	94.6	88.1	89.7	80.7

ANRS	92.0	83.9	94.4	87.7	73.3	72.7

HIVdb	94.3	95.0	94.5	86.2	87.6	79.7

Rega	95.9	86.0	94.0	92.2	88.3	73.8

SVM	98.7	98.1	98.4	99.2	96.5	97.5

ANN	98.2	98.4	98.7	98.3	96.5	97.0

Sparse dictionary	91.8	91.5	93.2	87.9	81.6	85.2

**Table 15:** Accuracy (%) compared to other methods for NNRTIs.

	NPV	DLV	EFV
HIV-grade	98.7	N/A	98.1

ANRS	94.8	N/A	97.9

HIVdb	98.4	N/A	98.7

Rega	98.6	96.8	98.7

SVM	98.2	98.3	99.1

ANN	98.3	98.6	98.6

Sparse dictionary	82.6	84.4	81.1

## Authors' contributions

All authors designed the experiments. XY and RWH designed the algorithms. XY implemented the algorithms and ran the predictions. All authors interpreted the results and wrote the manuscript. All authors read and approved the final manuscript.

## Declarations

The publication costs for this article were partly funded by the NIH-NIGMS grant U01GM062920.

This article has been published as part of *BMC Genomics *Volume 15 Supplement 5, 2014: Selected articles from the Third IEEE International Conference on Computational Advances in Bio and Medical Sciences (ICCABS 2013): Genomics. The full contents of the supplement are available online at http://www.biomedcentral.com/bmcgenomics/supplements/15/S5.
